# Effects of blood meal source on blood consumption and reproductive success of cat fleas, *Ctenocephalides felis*

**DOI:** 10.1371/journal.pntd.0011233

**Published:** 2023-04-13

**Authors:** Brittny N. Blakely, John Agnew, Charlotte Gard, Alvaro Romero

**Affiliations:** 1 Department of Entomology, Plant Pathology, and Weed Science, New Mexico State University, Las Cruces, New Mexico, United States of America; 2 Department of Economics, Applied Statistics, and International Business, New Mexico State University, Las Cruces, New Mexico, United States of America; National Institutes of Health, UNITED STATES

## Abstract

Cat fleas, small blood-feeding ectoparasites that feed on humans and animals, cause discomfort through their bites, and can transmit numerous diseases to animals and humans. Traditionally, fleas have been reared for research on live animals, but this process requires animal handling permits, inflicts discomfort on animals, and requires money and time to maintain the host animals. Although artificial membrane-based feeding systems have been implemented, these methods are not sustainable in the long term because they result in lower blood consumption and egg production than those with rearing on live hosts. To maximize these parameters, we tested blood from four hosts to determine the most suitable blood, on the basis of blood consumption and egg production. We also tested the effects of adding the phagostimulant adenosine-5´-triphosphate to the blood to maximize blood consumption. In 48 hours, fleas fed dog blood consumed the most blood, averaging 9.5 μL per flea, whereas fleas fed on cow, cat, or human blood consumed 8.3 μL, 5.7 μL, or 5.2 μL, respectively. Addition of 0.01 M and 0.1 M adenosine-5´-triphosphate to dog and cow blood did not enhance blood consumption. In a 1-week feeding period, the total egg production was also greatest in fleas fed dog blood, with females producing 129.5 eggs, whereas females on cat, human, and cow blood produced 97.2, 83.0, and 70.7 eggs, respectively. The observed results in dog blood indicate an improvement over previously reported results in cat fleas fed with an artificial feeding system. Improving the sustainability of rearing cat flea colonies without feeding on live animals will enable more humane and convenient production of this pest for scientific research.

## Author summary

Cat fleas cause discomfort through their bites, and can transmit numerous diseases to animals and humans. Traditionally, fleas have been reared for research on live animals, but this process requires animal handling permits, inflicts discomfort on animals, and is costly. Artificial feeding methods that warm blood and offer it through a membrane have been used but fleas often take small amount of blood and produce fewer eggs than those with rearing on live hosts. To maximize blood consumption and egg production, we offered fleas blood from four hosts to determine the most suitable blood for flea rearing. We also tested the effects of the addition of compounds that increase blood consumption (phagostimulants). Fleas fed dog blood consumed the most blood, followed by fleas fed on cow, cat, or human blood. Addition of phagostimulants did not enhance blood consumption. Fleas fed dog blood produced 129.5 eggs, whereas females on cat, human, and cow blood produced 97.2, 83.0, and 70.7 eggs, respectively. The observed results in dog blood indicate an improvement toward the production of cat fleas in the laboratory without feeding on live animals. This system will enable more humane and convenient production of this pest for scientific research.

## Introduction

Cat fleas, *Ctenocephalides felis* (Bouché), are blood-feeding insects that parasitize human, domestic animals, and many wild animals [1]. Biting activity by fleas can cause pruritic welts on the skin, and in some sensitive dogs and cats, these bites can trigger flea allergy dermatitis [2]. Cat fleas can also transmit numerous disease-causing bacteria to both humans and animals, including *Rickettsia typhi*, *R*. *felis*, and *Bartonella henselae* [2,3], as well as the intestinal parasite *Dipylidium caninum*, to dogs and cats [1]. Because fleas are the most common and important pest of dogs and cats worldwide, the control of these pests is a lucrative industry [4]. Pet owners spend more than US $15 billion annually on flea control products [5].

The importance of cat fleas is also reflected in the large amount of research being conducted on this pest. A topic search of literature in the Web of Science database in the past 20 years yielded more than 800 articles, most pertaining to the detection and transmission of diseases vectored by cat fleas and the efficacy of insecticide compounds. This high research activity requires many laboratory-reared cat fleas. Traditionally, flea colonies have been reared in laboratories on live animal hosts, but this process requires licensing with federal, state, and institutional agencies. In addition, live animal hosts require laboratory space for animal housing, money for purchasing and maintaining the animals, and person-hours for animal upkeep; moreover, they can cause discomfort to the hosts, owing to the fleas’ irritating bites and flea allergy dermatitis.

To eliminate the need for feeding fleas on live animal hosts, several artificial feeding systems have been implemented [6–9]. The earliest artificial feeding systems were developed to support the growth of the Oriental rat flea, *Xenopsylla cheopis* [8]. These systems consisted of fleas housed in a plastic tube with one end capped with a natural membrane made from animal skin or ox cecum and submerged in warmed animal blood. A system proposed by Wade and Georgi [9] houses cat fleas in plastic jars with meshed ceilings and delivers the blood meal with a custom glass feeder and an artificial membrane first described by Rutledge et al. [10] for feeding mosquitoes. Later, another feeding system replaced the glass Rutledge feeders with stainless steel cylinders, and parafilm as a feeding membrane, was marketed and sold as the “artificial dog” [11,12].

Although cat fleas can be reared in laboratories on an artificial feeding system, concerns have been raised regarding the sustainability of large-scale flea production with these systems, owing to the relatively low reproductive parameters. This concern has followed reports of diminished blood consumption and egg production in fleas fed on an *in vitro* system than on live cats [9,11,13]. Cat fleas raised on live cats consume three times more blood, and produce seven to eight times more eggs, than fleas fed bovine blood with an artificial feeding system [9,13]. These findings clearly indicate that aspects of artificial feeding systems can be improved upon to potentially increase blood ingestion and consequently egg production. In cat fleas, the blood from different hosts can influence consumption and egg production, depending on the animal from which the blood meal was obtained. For example, cat fleas fed on rats consume more blood and produce more eggs than those fed on mice [14]. Another study has indicated that cat fleas produce the most eggs when fed on pig blood rather than human, cow, sheep, or horse blood [15]. This trend can also be seen in other blood-feeding insects. Female bed bugs produce more eggs when fed on mice than domestic fowl or humans; however, no difference has been observed in the amount of blood consumed by these insects, thus suggesting that blood nutritional content may be a critical factor for oviposition [16]. Similarly, *Aedes aegypti* mosquitos fed human blood produce more eggs than those fed pig or sheep blood [17]. Moreover, consumption in artificial feeding systems has been found to be enhanced by the addition of phagostimulant compounds [18]. Although many blood-feeding insects, including oriental rat fleas, bed bugs, and mosquitoes, can be induced to feed on solutions containing adenosine-5´-triphosphate (ATP) [18–20], previous studies with cat fleas have shown that ATP at 0.005 M does not enhance engorgement when added to blood in artificial feeding systems [9,21]. This finding may be explained by the rapid depletion of ATP in the stored blood [22] that remains warmed in the artificial feeding system for hours.

The purpose of this study was to identify the most suitable blood meal by comparing the blood consumption and egg production of cat fleas fed blood from four species of host animals, and to determine whether higher concentrations of the phagostimulant ATP might induce greater blood consumption than previously reported. Optimizing the conditions of the artificial feeding system for cat fleas could improve reproductive parameters and the long-term production of fleas in laboratory settings.

## Materials and methods

### Fleas

Newly emerged cat fleas (1–2 days post-emergence) reared on an artificial feeding system with sheep blood was purchased from Elward Labs (Soquel, CA). Fleas were used in bioassays at 3–4 days post-emergence, and were kept in a growth chamber (Thermo Scientific, 818, Marietta, OH) at 24°C, 75% RH, with a 12:12 (L:D) h. To facilitate handling, the fleas were anesthetized with a Flowbuddy CO_2_ anesthesia system (Genesee Scientific, San Diego, CA).

### Blood

Dog, cow, cat, and human blood in 3.8% sodium citrate was purchased from Biochemed Services (Winchester, VA) and used within 10 days after collection. In experiments using the phagostimulant ATP, adenosine 5´-triphosphate dipotassium salt hydrate (MilliporeSigma, St. Louis, MO) was mixed directly into dog or cow blood to create 0.1 M and 0.01 M solutions. During the experiments, all blood meals were replaced with fresh blood every 24 h.

### Artificial feeding system

Blood meals were delivered to the fleas with glass feeders similar to those described by Rutledge et al. [10]. The glass feeders were 40 mm in diameter and were manufactured by Prism Research Glass (Raleigh, NC). A piece of Buddy grafting tape (Aglis, Yame City, Japan) was stretched across the bottom of the feeder to contain 2 ml of blood in the feeder and allowing the fleas to pierce the membrane with their mouthparts and feed on the blood. The feeders were placed in clamps attached to laboratory stands, and flea containers were held underneath the feeders against the membrane with laboratory scissor jacks. The blood was warmed to 34°C with a Lab Companion warm water circulator (Jeio Tech, CW-05G, Billerica, MA).

### Feeding cages

**Fig 1:**
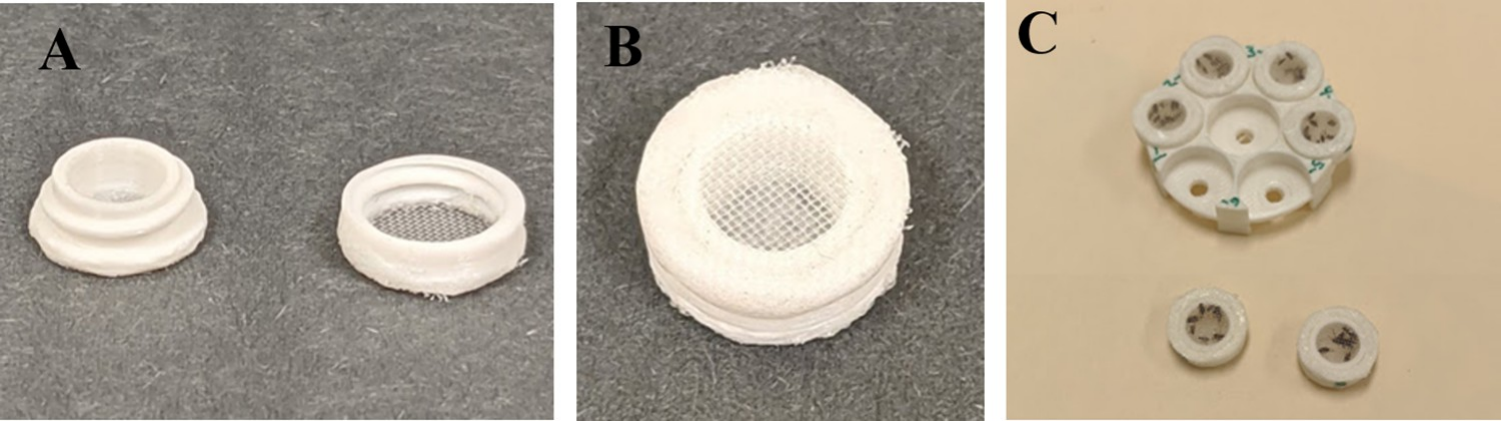
Custom feeding cages. (A) Subsample cage, bottom and lid. (B) Assembled subsample cage. (C) Cage holder with subsample cages placed in wells.

To facilitate subsampling within the experiments, we used custom feeding cages. Each individual subsample feeding cage (12 mm wide × 5 mm high) comprised a top and bottom that screwed together (Fig 1A and 1B). The top and bottom pieces contained a hole 6 mm in diameter. Nylon plankton netting with 500 μm pores (Bioquip, Rancho Dominguez, CA) was glued across the hole in the top, and nylon mesh with 30 μm pores (Amazon, Seattle, WA) was glued across the hole of each bottom piece with methylene chloride glue (Sigma Aldrich, St. Louis, MO). Subsample holders (35 mm wide × 9 mm high) were used to keep all subsamples together and level, to provide an equal opportunity for each subsample to take a blood meal (Fig 1C). Each holder had six wells (13 mm wide × 2 mm high), which were spaced around the perimeter and could hold a subsample cage, and a 7^th^ space for one subsample cage in the middle, which was not used. The subsample cages and holders were designed with Blender (Blender Foundation, Amsterdam, Netherlands), sliced and prepared with Cura (Ultimaker, Utrecht, Netherlands), and 3D printed on an Ender 3 printer (Creality, Shenzen, China) with white PLA filament (MatterHackers, Lake Forest, CA).

### Effects of host animal species on blood consumption

To test the effects of dog, cow, cat, or human blood on blood consumption, we offered cat fleas blood meals for 48 h. Fresh blood was given at 0 h and 24 h. At the end of the 48 h, the adult fleas and feces from each subsample were placed into microcetrifuge tubes and frozen at -18° C for the estimation of blood consumption. Cat fleas were randomly assigned to test groups comprising three subsamples of ten fleas (1:1 sex ratio), and the experiment was replicated twice.

### Effect of the addition of ATP on blood consumption

To test whether the addition of ATP increases blood consumption, we offered cat fleas blood meals with 0.01 M ATP or 0.1 M ATP. Intact blood (with no added ATP) was used as control. Fresh blood + ATP was offered at 0 h and 24 h. At the end of 48 h, the adult fleas and the feces from each subsample were placed into microcentrifuge tubes and frozen at -18° C for the estimation of blood consumption. For this experiment, cat fleas were randomly assigned to test groups comprising three subsamples of nine female fleas, and the experiment was replicated three times.

### Effects of 1-week blood feeding on egg production, larval hatching, and adult mortality

To test the effects of blood from different species of animal hosts on egg production, we offered cat fleas blood meals for 1 week. Every 24 h, fresh blood was given, the number of dead adults was counted, and eggs were collected to determine egg production and larval hatching. Cat fleas were randomly assigned to test groups comprising three subsamples of ten fleas (1:1 sex ratio), and the experiment was replicated three times.

### Quantification of blood consumption

Blood consumption was quantified according to the protocols outlined by Kern et al. [23] and McCoy [24]. In brief, 1.5 mL of de-ionized water was added to each tube containing feces, and 0.5 mL was added to tubes containing fleas. The tubes containing feces were agitated to dissolve the feces, whereas the fleas were macerated in their tubes. The fecal solution was transferred into 12 mL glass vials containing 3.5 mL deionized water, and the flea bodies and their solutions were transferred into 12 mL glass vials containing 4.5 mL deionized water. Drabkin’s solution was prepared by mixture of one vial of Drabkin’s reagent (MilliporeSigma, St. Louis, MO) with 1 L of deionized water and 0.5 mL of Brij L23 Solution (MilliporeSigma, St. Louis, MO). Subsequently, 5 mL of prepared Drabkin’s solution was added to each 12 mL vial, and each vial was vortexed for 1 minute and allowed to stand for 30 minutes. The contents of each vial were then filtered through Whatman filter paper #1 (Whatman, 1001–090, Maidstone, United Kingdom) into clean 12 mL vials, and the optical density (OD) of 1 mL of each sample was measured at 540 nm with a spectrophotometer (Beckman, DU 530, San Jose, CA). The volume of blood in each sample was then estimated by comparison of their ODs to calibration curves created for each blood type.

To create these calibration curves, we added 2, 5, 10, 20, or 40 μL of blood to 12 mL glass vials containing 5 mL each of deionized water and Drabkin’s solution. Each sample was then processed, and the OD was read as described above. This process was repeated three times. Each OD was then plotted on the basis of the volume of blood in the sample, and a linear regression was calculated according to those data points. This process was repeated for each of the four blood types, and the R^2^ values were 0.96, 0.98, 0.99, and 0.97 for dog, cow, cat, and human blood, respectively (S1–S4 Figs).

### Quantification of egg production and larval hatching

The eggs and feces produced in each cage were transferred daily into 2 mL microcentrifuge tubes (Sigma Aldrich, St. Louis, MO), each with an 8 mm hole cut in the top and 500 μm nylon plankton netting (Bioquip, Rancho Dominguez, CA) attached with hot glue. These microcentrifuge tubes were kept in the same growth chamber at 24°C, 75% RH, with a 12:12 (L:D) h, and were verified every day for larval hatching. As larvae hatched, they were counted and individually transferred on the tip of a dissecting needle to plastic screw-top jars 38 mm wide × 20 mm high (Hobby Lobby, Oklahoma City, OK) containing previously described flea larval growth medium [25]. The lids and bottoms of these jars had a 30 mm hole cut into them and nylon mesh with 30 μm pores attached with methylene chloride glue. After 2 weeks, the eggs and feces were placed into a funnel lined with Whatman filter paper #1, and water was poured through the funnel to dissolve the feces. After all feces were washed away, eggs were counted under a dissecting microscope (Leica, Stereozoom s9i, Wetzlar, Germany) to estimate total egg production. Larval hatching was calculated by division of the number of larvae hatched by the total number of eggs produced.

### Data analysis

Statistical analysis was conducted in SAS v 9.4 (SAS Institute, Cary, NC), and graphs were created with Sigma Plot v 10.0 (Systat, San Jose, CA). One-way analysis of variance (ANOVA) with subsampling was used to analyze the 48 h blood consumption data from the host species assays, and the 1-week mortality, 1-week average daily egg production, and 1-week larval hatching data from the host species assay. In all these analyses, Fisher’s least significant difference post hoc tests were performed to distinguish differences between the means. One-way ANOVA with subsampling was also used to analyze the 48 h blood consumption data from the ATP study. However, cube root and natural log transformations of dog and cow blood data, respectively, were necessary before ANOVA, and upper-tailed Dunnett’s tests were used as post hoc with 0 M ATP as the control. One-way ANOVA with repeated measures was used to analyze the 1-week cumulative egg production data. A Bonferroni adjustment based on the number of comparisons per day was applied to distinguish the differences in the daily egg cumulative means.

## Results

### Blood consumption

**Fig 2:**
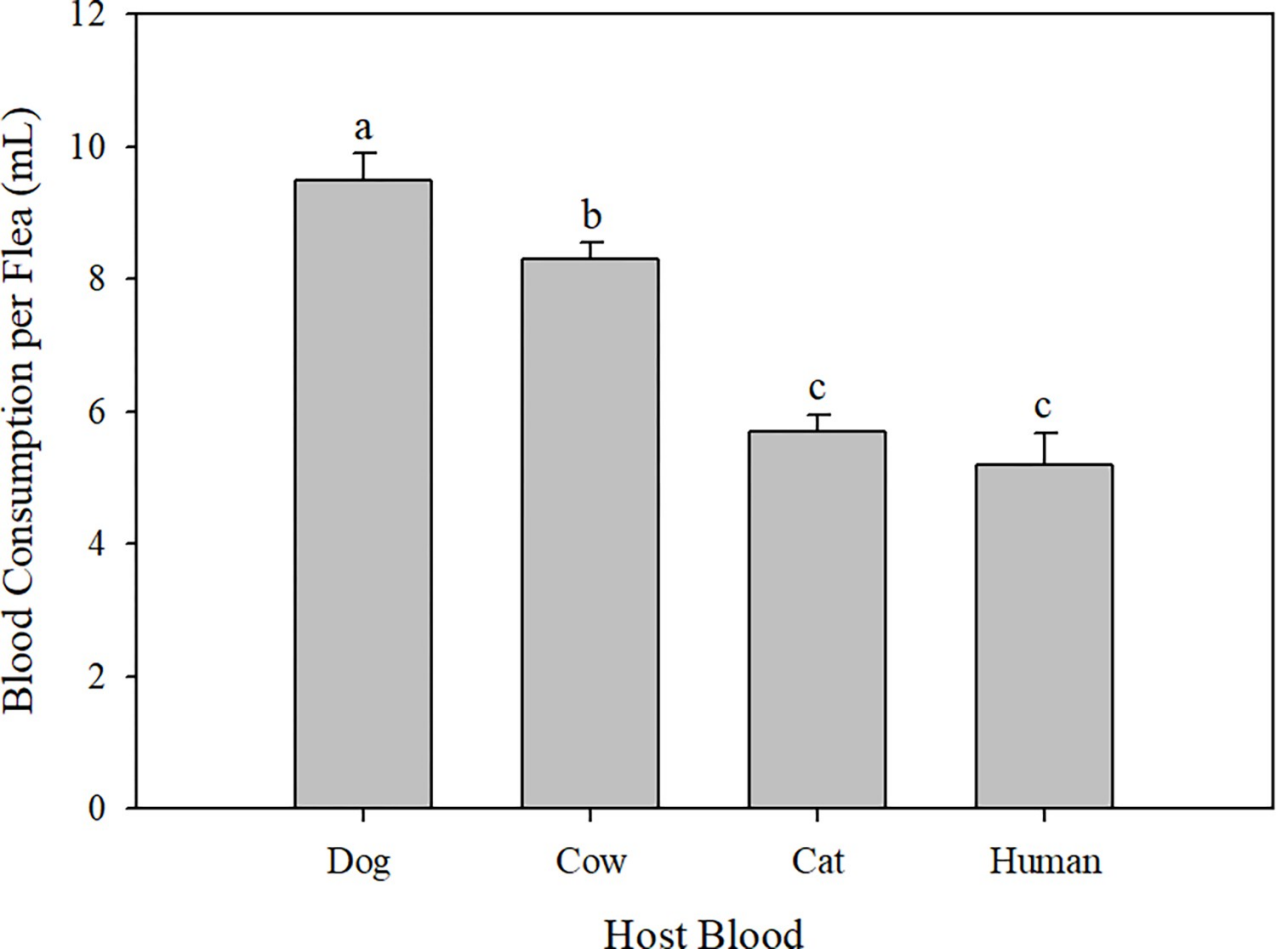
48-hour blood consumption of cat fleas fed blood from different host species. Different letters above the bars indicate significant differences (One-way ANOVA with subsampling; Fisher’s least significant difference test; p < 0.05).

After 48 hours of feeding, dog blood was the most consumed blood by fleas (9.49 μl per flea) (F_3.4_ = 52.24, p = 0.001) (Fig 2), followed by cow blood (8.30 μl), cat blood (5.67 μl), and human blood (5.2 μl). Cat fleas fed on cow blood ingested significantly more blood than cat fleas fed on cat or human blood (p < 0.05).

### Effect of the addition of ATP on blood consumption

**Fig 3:**
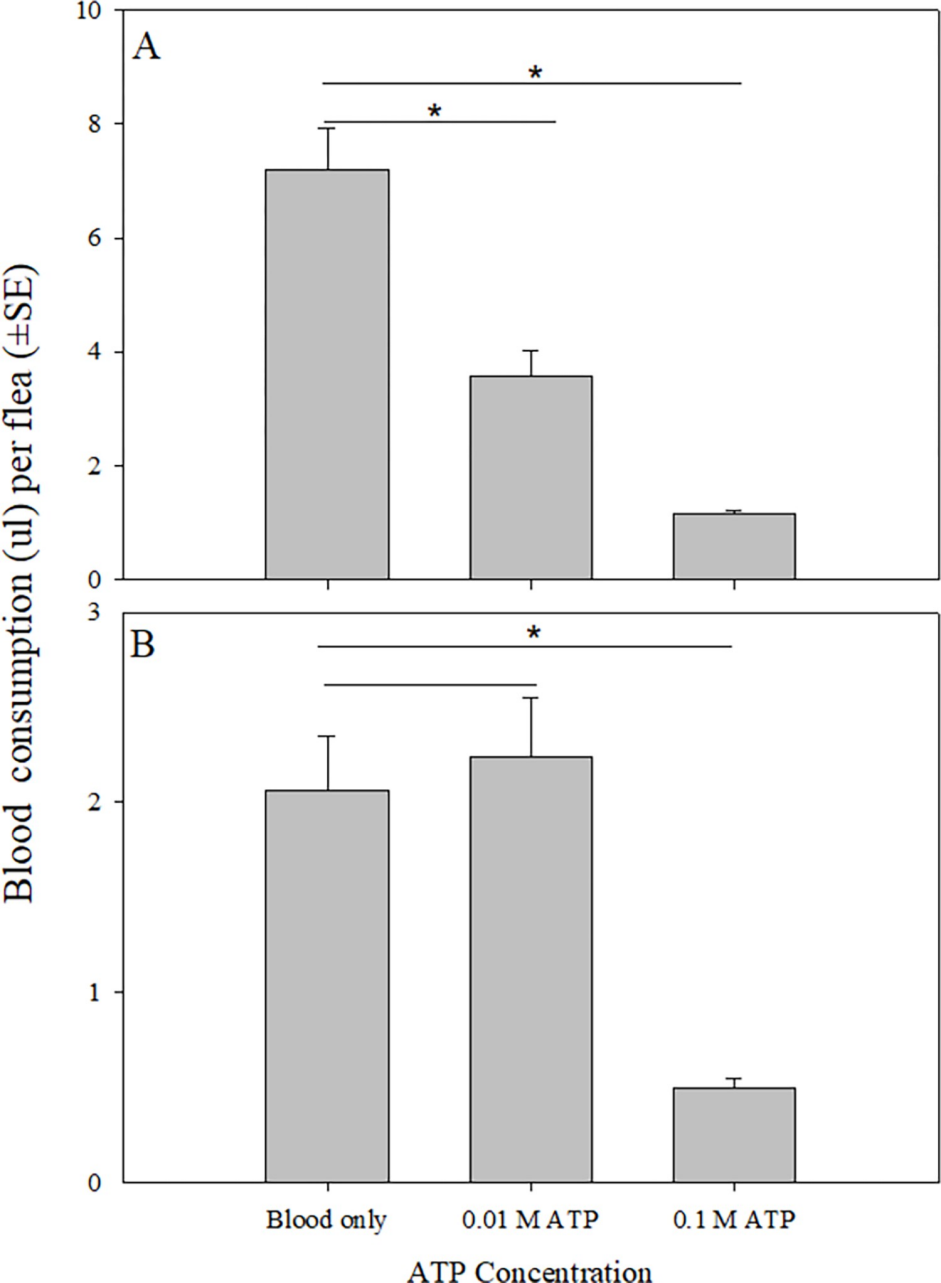
**48-hour blood consumption of cat fleas fed dog (A) and cow (B) blood with added ATP.** blood only, blood with 0.01 M added ATP, and blood with 0.1 M added ATP. Asterisk indicates parameter values significantly different from those in the control group (blood only) (One-way ANOVA with subsampling; upper-tailed Dunnett’s test; p < 0.05).

Addition of ATP to blood reduced the volume of dog blood consumed per flea when compared to control groups (F2,5 = 195.08, p < 0.001). Groups of fleas fed dog blood with either 0.01 M or 0.1 M ATP consumed two times and six times less blood than groups of fleas fed dog blood without ATP (Fig 3A). Similarly, when added to cow blood, ATP reduced the volume of blood consumed per cat flea (F2,6 = 21.01, p = 0.002). Whereas fleas fed cow blood with 0.01 M ATP consumed a similar amount of blood to fleas fed cow blood with no ATP, fleas fed ATP at the higher concentration consumed four times less blood than those in the control group without ATP (Fig 3B).

### One-week egg production

After 1 week of feeding, groups of 45 female fleas fed on dog blood produced a total of 5,431 eggs when fed on dog blood. This number was 116.1% more than those produced by fleas fed on cow blood (2,510 eggs), 52.4% more than those produced by fleas fed on human blood (3,563 eggs), and 45.0% more those produced by fleas fed on cat blood (3,747 eggs). On a daily basis, each female cat flea fed on dog blood for 1 week produced more eggs than those fed on any other animal’s blood (F_3,8_ = 7.54, p = 0.01) (Table 1). Females fed dog blood produced 59.1% more eggs on average than females fed cow blood, 40.7% more eggs than females fed human blood, and 30.7% more eggs than females fed cat blood.

**Table 1:** Daily egg production, hatching, and mortality rates of cat fleas fed blood of different host species during 1 week.

	Egg production^1^	% Larval hatching^1^	% Mortality^1^
	Eggs/female/day		
Dog	18.3 (1.8)^a^	41.1 (4.2)^a^	5.6 (0.5)^a^
Cow	11.5 (4.9)^b^	38.9 1.6)^a^	21.7 (3.4)^a^
Cat	14.0 (7.6)^b^	25.2 (1.4)^a^	11.3 (1.0)^a^
Human	13.0 (2.1)^b^	36.9 (1.7)^a^	13.9 (1.4)^a^

^1^ Different superscripts represent significant differences between values within a column (One-way ANOVA with subsampling; Fisher’s least significant difference test; p < 0.05).

‡ Data represents the mean (SE)

**Fig 4:**
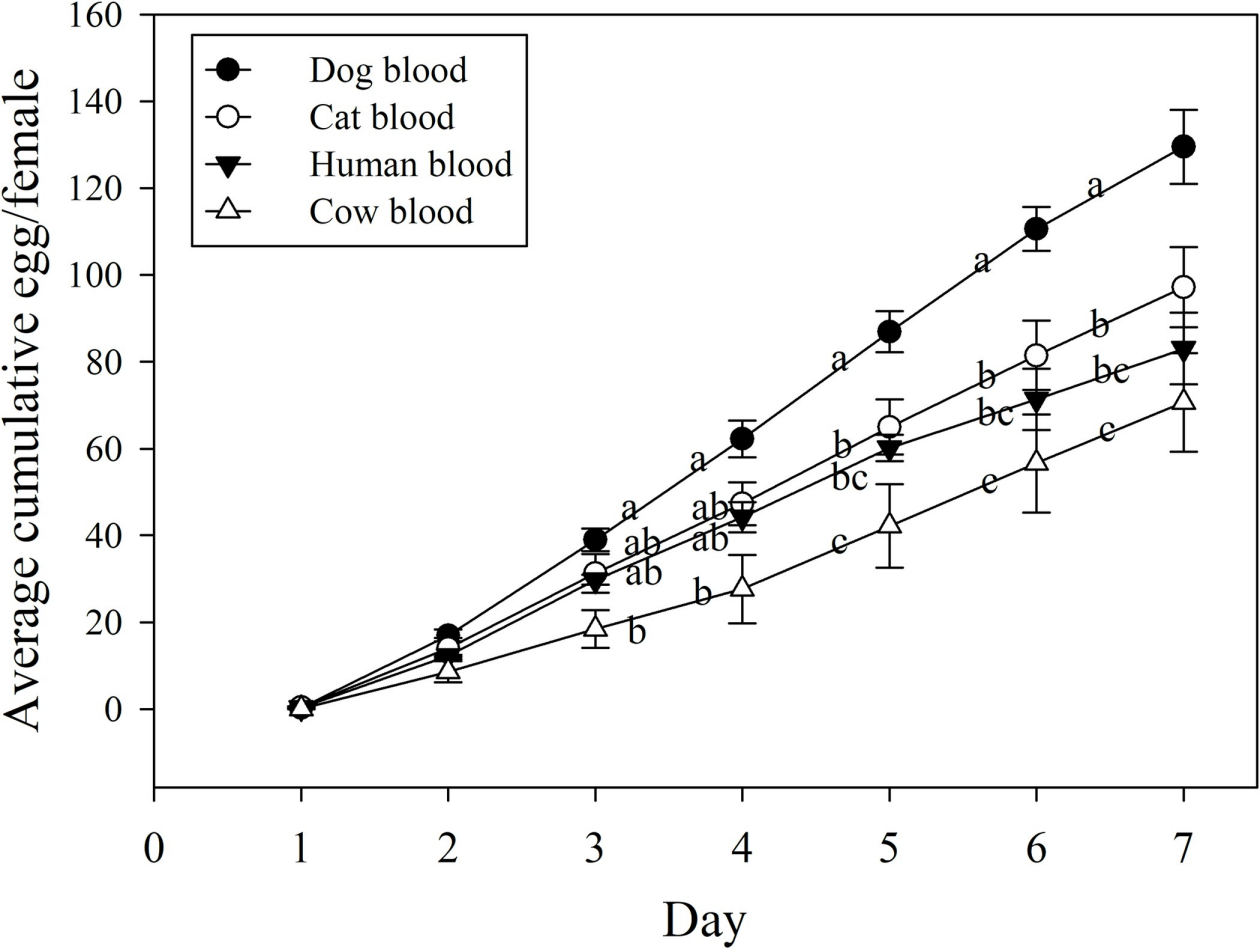
Daily cumulative egg production of cat fleas fed blood of different host species. Different letters represent significant differences between means within a day (One-way ANOVA with repeated measures; Bonferroni adjustment; p < 0.05).

No differences were observed in the hatching rates of eggs produced by fleas fed blood from different host animals (F_3,8_ = 1.10, p = .402) or in the percentage mortality of adult fleas fed blood from different host animals (F_3,7_ = 1.42, p = 0.316) (Table 1). Cumulative daily egg production per female also differed across blood types (F_18,48_ = 4.71, p < .0001). During the 1-week feeding period, fleas fed dog blood began to produce significantly more eggs than fleas fed cow blood on day 3, and by day 5, they produced the most cumulative eggs per female (Fig 4). This trend continued throughout the rest of the week: fleas fed dog blood laid an average of 130 eggs per female by the end of the week, a number 51.8%, 29.9%, or 14.5% greater than those laid by females fed on cat, cow, or human blood (Fig 4).

## Discussion

In this study, cat fleas fed dog blood consumed more blood than fleas fed on blood from other hosts. A comparison with past studies that used artificial feeding systems shows that this represents an improvement in blood consumption. Hinkle et al. [13] have reported flea consumption of an average of 2.3 μL of cow blood in a day, whereas our fleas consumed 4.7 μL (108% increase) of dog blood in a day. However, the observed consumption of dog blood remained below the daily blood consumption of cat fleas fed on live cats, which has been reported to be 7.0–15.4 μL [13,24,26].

The observed differences in blood consumption of fleas fed from four animal hosts indicated differences in the acceptability of the blood to fleas. Blood phagostimulants are a major factor that insects rely on to stimulate feeding and engorgement [18]. In most blood-feeding insects, the major phagostimulatory compounds are ATP and adenosine diphosphate (ADP), which are associated primarily with the cellular fraction of host blood, including red blood cells (RBCs) and platelets [20,27]. Therefore, blood with higher hematocrit may contain more RBC associated phagostimulants [28,29]. This possibility could at least partially explain why dog blood, which has the highest hematocrit (range 35%–57%) [30] among the species tested, was the most consumed blood.

In artificial feeding systems, blood ingestion by insects can be affected by the depletion of ATP in the blood remaining in the feeders. Blood maintained in long-term cold storage conditions or overnight at room temperature can have diminished ATP concentrations [22]. To circumvent this decrease, we increased the ATP in the blood meals to 10 and 100 times the physiological concentration of ATP (0.001 M) [31] in an effort to maintain effective levels during the feeding time of the fleas. However, ATP addition did not increase blood consumption. Moreover, fleas fed blood with higher concentrations of ATP displayed high mortality (dog: 98%; cow: 100%). When the blood meals were replaced after 24 hours of feeding, the blood samples mixed with the highest ATP concentration tested (0.1 M ATP) were completely coagulated in the feeders. Although ATP has not been reported to interact with sodium citrate, ATP inhibits the antihemostatic properties of another common anticoagulant, heparin, both *in vivo* and *in vitro* [32]. To mitigate any potential ATP/anticoagulant interactions, future studies involving ATP might consider the use of defibrinated blood rather than blood treated with chemical anticoagulants. However, the effect of defibrination on egg production should also be investigated. Additionally, replacing blood meals that include ATP more frequently may be required to improve blood ingestion by cat fleas.

Cat fleas are anautogenous and must feed on blood from a vertebrate host to produce eggs (68). Blood consumption and egg production are generally positively correlated [14,33–35]. Although we could not directly correlate blood consumption with egg production over a feeding period of 1 week, results from the 2-day blood consumption might allow us to draw conclusions regarding the effects of blood ingestion on egg production. For example, the highest number of eggs were produced by fleas fed for 1 week on dog blood, which was the most consumed blood in the 2-day consumption experiment. However, evidence has suggested that the blood nutritional qualities of certain host animals can influence egg production [33]. For example, in our study, consumption of cat and human blood, which were the least consumed blood by female cat fleas, was associated with more eggs than consumption of cow blood, the second most consumed blood (Figs 2 and 4). Blood nutrients that are directly associated with egg formation in hematophagous insects include proteins and micronutrients such as free amino acids [36–38]. Although protein can be found in both blood fractions, most protein is located in RBCs as hemoglobin [30]. Hemoglobin provides most of the eight essential amino acids necessary for egg production, and isoleucine is particularly important for oogenesis [38,39]. The low level of isoleucine in human blood [39] may account for the lower egg production in fleas fed human blood rather than dog blood. Because hemoglobin is crucial for egg production in anautogenous insects, such as mosquitos [40], the number of eggs produced should correlate with the blood’s hematocrit. In our data, this correlation appeared to be present: fleas fed dog blood, which has the highest reported hematocrit (range 35–57%), produced more eggs than fleas fed human, cat, or cow blood (reported hematocrit ranges of 36–41%, 30–45%, and 24–46%, respectively) [30]. This argument is also supported by the tendency of dog blood to contain more hemoglobin in RBCs than the blood from the other animals studied [30]. Moreover, plasmatic proteins may be the source of protein for egg production in certain hematophagous insects [41]. Therefore, differences in plasmatic proteins among the blood samples tested in our study may explain differences observed in flea oviposition. More research is needed to better understand this relationship, and to determine which blood compounds, or combinations of compounds, are most important in egg production and thus the development of artificial diets for fleas.

Overall, average daily egg production of females fed on dog blood was higher than those reported in other studies that have used artificial feeding systems. Females fed on dog blood produced an average of 18.3 eggs per day, whereas published studies have reported 13.7 eggs per day on pig blood [15], 2.3–5.7 eggs per day on cow blood [9,14,42], and 3.6 eggs per day on human blood [11].

Compared with female fleas fed on live cats, our female fleas fed on dog blood had an average daily egg production similar to the previously reported 13.5–29.1 eggs [9,13,26,43–45]. Curves of cumulative egg production of cat fleas showed a sustained increase in eggs throughout the 7-day oviposition. Given the steep slope of this curve, further feeding of fleas beyond 7 days may be very likely to increase total oviposition and therefore increase progeny.

No difference was observed in the percentage of larval hatching or adult mortality between groups of fleas fed for 1 week on blood from different host animals; however, the larval hatching rate observed in our study, 25.2–41.1%, was lower than those in all but one value reported in cat fleas fed on artificial feeding systems in prior studies, 14.3–86% [9,11,42], and was lower than the reported larval hatching rates of cat fleas fed on live cats, 50.4–85% [9,11]. This observed decrease in larval hatching might be explained by how the eggs, feces, and larvae were housed. In our study, the collected feces and unhatched eggs were stored in 2 mL microcentrifuge tubes with 10 mm diameter. These tubes were verified daily for hatching, and any larvae were counted and transferred to another container with larval growth medium. Even with daily inspection, some egg tubes became overcrowded with larvae, thus possibly leading to cannibalism of the larvae before they were transferred. In future studies, flea eggs and larvae should be housed in larger containers to ensure that the hatching rate is not diminished due to overcrowding and cannibalism.

Although our results show an improvement in the *in vitro* blood consumption and egg production of cat fleas, additional research remains needed. Host-associated cues are known to help blood-feeding insects detect and select their hosts [18]. In an artificial feeding system, the host, and therefore most of these cues, are absent, except for heat from the blood meal and skin simulation from the feeding membrane. Host odor and tactile stimulation can be added to artificial feeding systems by including hair from a host animal in the flea cages [9,11]. *in vitro* blood consumption could be further improved by supplementing host cues such as CO_2_ or using alternative feeding membranes that get impregnated with host body odors into these *in vitro* protocols, thereby creating a more natural feeding environment.

Improved egg production in cat fleas raised on an artificial feeding system could also be achieved by altering the sex ratio of females and males in the feeding cages. In a laboratory setting on an artificial feeding system, the sex ratio for optimum egg production has been reported to be 4:1 (F:M), and a 1:1 sex ratio is second best [15]. Owing to the small number of individuals in our test groups, we used an equal sex ratio rather than a female-biased ratio to ensure the presence of adequate males for insemination in the event of mortality. However, in attempts to raise cat fleas *en masse*, to fully maximize egg production on an artificial feeding system, a higher F:M ratio, closer to that reported by Kernif and colleagues [15], is recommended.

In our study, we determined that dog blood is a suitable blood for raising cat fleas on an artificial feeding system, and it performed better than cow, cat and human blood in terms of blood consumption and egg production. Additional research is needed to determine whether dog blood is a suitable nutritional source for completion of the flea’s life, in which parameters such as larval hatching and adult emergence are also optimized. Similarly, assessment of the overall fitness parameters of fleas, such as size, weight, and reproductive parameters [46], should be evaluated when fleas are reared for multiple generations.

Finally, although an artificial feeding system provides a better alternative to live animal hosts, *in vitro* systems still require animal blood, which is expensive and has a short 2-week shelf life. Recent mosquito research has formulated completely blood-free diets that still allow females to produce eggs [47]. Further research on the specific blood properties that influence engorgement and egg production in cat fleas could be performed to enable the formulation of blood-free diets that allow for the successful reproduction of cat fleas without blood.

## Supporting information

S1 Fig Calibration curve for dog blood. (TIF)

S2 Fig Calibration curve for cow blood. (TIF)

S3 Fig Calibration curve for cat blood. (TIF)

S4 Fig Calibration curve for human blood. (TIF)
