# Supplementary figures and images for: Effects of blood meal source on blood consumption and reproductive success of cat fleas, Ctenocephalides felis
Source: PLoS Negl Trop Dis. 2023 Apr 13;17(4):e0011233. doi: 10.1371/journal.pntd.0011233 (PMC10101638; doi:10.1371/journal.pntd.0011233)

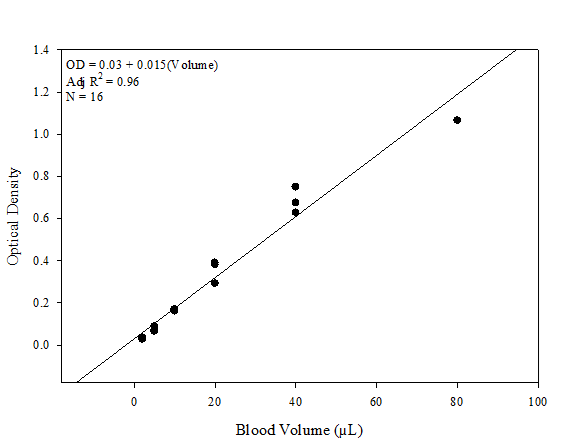

Supplement: S1 Fig — (TIF) [file pntd.0011233.s001.tif]

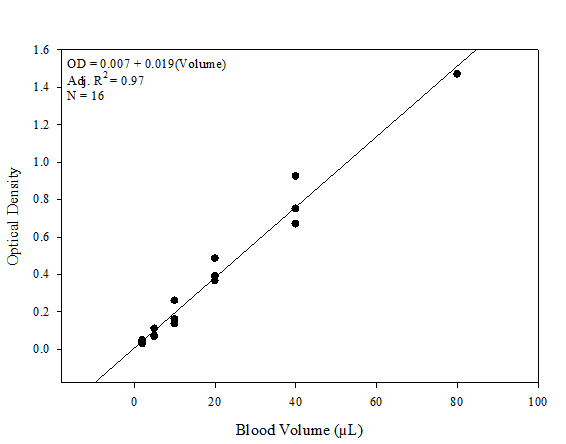

Supplement: S2 Fig — (TIF) [file pntd.0011233.s002.tif]

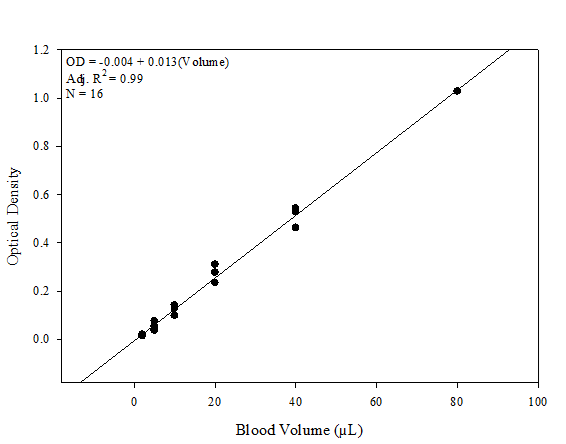

Supplement: S3 Fig — (TIF) [file pntd.0011233.s003.tif]

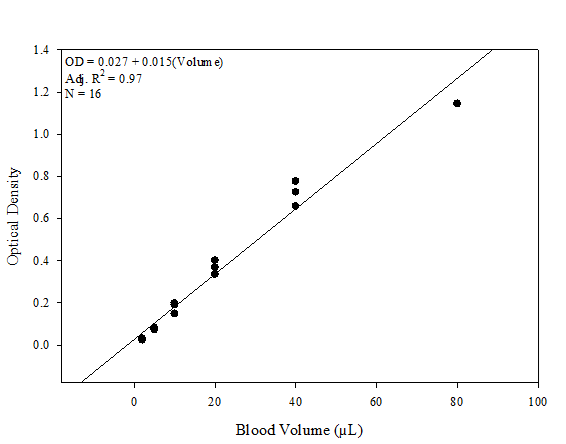

Supplement: S4 Fig — (TIF) [file pntd.0011233.s004.tif]
